# Validation of the European Portuguese Version of the Yale Pharyngeal Severity Rating Scale

**DOI:** 10.1007/s00455-024-10731-0

**Published:** 2024-07-26

**Authors:** Isabel Silva-Carvalho, Adriana Martins, Susana Vaz Freitas, Laetitia Teixeira, Luís Meireles, Isabel Pedroto

**Affiliations:** 1 Neurosciences Department, ENT, Centro Hospitalar Universitário de Santo António, Instituto de Ciências Biomédicas Abel Salazar, Largo Professor Abel Salazar, Porto, 4099-001 Portugal; 2 Surgery Department, ENT, Unidade Local de Saúde Gaia e Espinho– Centro de Reabilitação do Norte, Avenida Infante Sagres, 349, Valadares, 4405-565 Portugal; 3 Neurosciences Department, ENT, Centro Hospitalar Universitário de Santo António, ESS.IPP.LIAAD – INESC TEC, Largo Professor Abel Salazar, Porto, 4099-001 Portugal; 4 Instituto de Ciências Biomédicas Abel Salazar, Rua Jorge de Viterbo Ferreira, 228, Porto, 4050-313 Portugal; 5 Medicine Department, Gastroenterology, ,Centro Hospitalar Universitário de Santo António, , Instituto de Ciências Abel Salazar, Largo Professor Abel Salazar, Porto, 4099-001 Portugal

**Keywords:** Pharyngeal residue, Deglutition disorders, Therapy, Fiberoptic endoscopic evaluation of swallowing

## Abstract

This study aimed to validate the Yale Pharyngeal Residue Severity Rating Scale’s European Portuguese version and investigate the impact of rater experience. The scale measures the severity of residue in the vallecula and pyriform sinus. Ninety Fiberoptic Endoscopic Evaluation of Swallowing images were selected after consensus and proposed to 13 raters who were asked to assess the severity of pharyngeal residue (PR) in each image in two moments with an interval of two weeks. The raters were divided by years of experience conducting the Fiberoptic Endoscopic Evaluation of Swallowing and in experience using severity scales for residues. Construct validity, inter-rater, and intra-rater reliability were assessed by kappa statistics. The original English scale was translated into European Portuguese using a forward–backward method for validation. The scale reliability was strong, with an elevated intra-rater internal consistency for vallecula (Cronbach’s alpha = 0.982) and pyriform sinus (Cronbach’s alpha = 0.922). Inter-rater reliability for raters was equally significant and high for vallecula (0.613 for first assessment and 0.604 for second assessment) and pyriform sinus (0.558 for first assessment and 0.509 for second assessment) or for raters with experience using Yale Pharyngeal Severity Rating Scale (vallecula with 0.832 for first assessment and 0.717 for second assessment and pyriform sinus with 0.856 for first assessment and 0.714 for second assessment).The European Portuguese version of the Yale Pharyngeal Severity Rating Scale is a valid, reliable instrument for scoring the location and severity of pharyngeal residue in the context of fiberoptic endoscopic evaluation of swallowing.

## Introduction

The pharyngeal residue (PR), defined as pre-swallow secretions and post-swallow food residue in the pharynx not entirely cleared by a swallow, is a clinical predictor of post-swallow aspiration and impaired efficacy [1]. PR occurs in either the vallecula or the pyriform sinuses [2]. The PR is an important indicator used in evaluating and planning the treatment of patients with dysphagia and monitoring the results of swallowing therapy [3]. Fiberoptic endoscopic evaluation of swallowing (FEES) and videofluoroscopic swallow study (VFSS) are established gold standard methods for the diagnosis and management of dysphagia [4, 5]. FEES is an evaluation method that allows both the larynx and the pharynx to be seen simultaneously with flexible endoscopy. Regarding PR identification, FEES seems superior to VFSS but leads to more severe impressions. It is a practical and reliable method that includes the evaluation of the pharyngeal phase of swallowing with a food bolus with a certain fluidity and color that contrasts with the mucosa [6, 7].

PR is evaluated by examining how much food or liquid remains in the vallecula and pyriform sinuses after swallowing. Assessment scales differ in the number of levels and descriptions, including percentage filling, visual analog scale scores, and anatomical landmarks. Scoring timing also varies, either after the first swallow or after clearing swallows [8–13]. Also, measurement scales to score saliva pooling (sit, amount, and response to saliva pooling) have been developed [1, 14]. The Yale Pharyngeal Residue Severity Rating Scale (YPRSRS), an anatomically defined and image-based tool, is a 5-point ordinal scale with a separate residue severity score for the vallecula and the pyriform sinuses; it is a validated, reliable tool to assess PR based on FEES [11, 12, 15]. To date, YPRSRS has already been translated into German, Turkish, and Italian languages [3, 16, 17] and has been reported to be a reliable ordinal scale with high validity. In 2022, Rocca et al. showed satisfactory reliability and construct validity in FEES videos, not only images [18]. In Portugal, the first days of instrumental evaluation of the oropharyngeal dysphagia (OD) are now beginning to be given. The validation of PR severity scales is an added value for evaluating and studying OD.

For translation, the authors were based on the guidelines for translation and cultural and linguistic adaptation proposed by Beaton et al. [19].

This study aimed to develop a Portuguese translation of YPRSRS and evaluate its construct validity, inter-rater, and intra-rater reliability. In addition, the authors investigated the impact of experience of rater.

## Methods

### Ethics

The study was approved by Centro Hospitalar e Universitário de Santo António (CHUdSA) and performed according to the Declaration of Helsinki.

### Design of the Original Scale

The rating table in the YPRSRS assesses two anatomical regions: vallecula and pyriform sinus. Ratings range from none to severe: none (0%), trace (1–5%), mild (5–25%), moderate (25–50%), and severe (> 50%).

### Translation Process

YPRSRS was translated into Portuguese by two English-Portuguese bilingual translators, and the Portuguese version was translated back into English by two professional translators qualified to assess accuracy. The translation was revised according to professional terms by three clinical specialists with experience in the study of swallowing who spoke foreign languages.

### Validation Process

The study design of this validation was oriented closely to the study design of the scale’s original version, based on the hierarchical categorization of images using the European Portuguese version of the scale.

### Image Selection Process

During the image selection process, 150 images were obtained from a FEES database to represent different degrees of severity of blue-colored residues. Each image was displayed separately with a scaling of 720 × 476 pixels. The images included residues with different food consistencies according to the International Dysphagia Diet Standardization Initiative: thin liquid (level 0), slightly thickened liquid (level 2), moderately thickened liquid (level 3), extremely thickened liquid (level 4), regular solid (level 7). The FEES procedures used a Rino Pharyngo Laryngo 3.7 × 30 cm Cat KST-11101RP1 Karl Storz fibroscope. As there is no valid gold standard in European Portuguese, an expert in FEES with over ten years of experience established reference values for residue severity ratings.

To ensure construct validity calculation, the 150 images were independently categorized by two experts using a scale in the first step of the selection process. Only the images with consistent evaluations from experts (n = 90) were included, while those with divergent evaluations were excluded (n = 60).

In the second step, the images were divided by location (45 vallecula and 45 pyriform sinuses). This division ensured the representativeness of each severity level.

### Raters

A total of 13 raters participated in the study: 12 otorhinolaryngologists and one SLT with experience in FEES; 11 never used the scale, and two have used it in their clinical practice for over five years.

### Data Collection

Ratings of the 90 randomized color images (45 vallecula and 45 pyriform sinus) were given via an online platform, and a second rating was carried out at an interval of two weeks. A brief scale explanation was given, and a document containing the original images and severity classification of residue was provided.

### Statistical Analysis

For the intra-rater agreement, the first and second evaluation scores were analyzed using the intra-class correlation coefficient (ICC). Individual and average ICC coefficients were reported with 95% confidence intervals and p-values. The first and second measurements were also analyzed using the quadratic weighted Kappa coefficient, according to the judges. The lowest and highest measures were reported. Agreement analyses were performed for the overall sample, for each anatomic part (vallecula and pyriform sinus) and level of experience (> 5 vs. ≤ 5 years) status.

For the inter-rater agreement analysis, Fleiss’s kappa was calculated. The inter-rater agreement analyses were also performed for the overall sample and each anatomic part (vallecula and pyriform sinus), measurement (first and second), and level of experience on FEES (> 5 vs. ≤ 5 years) status.

For the inter-rater agreement analysis considering the two judges with experience on residue severity scales, Cohen’s Kappa was calculated for each anatomic part (vallecula and pyriform sinus) and measurement (first and second).

A significance level of 0.05 was considered. All analyses were performed using SPSS 28.0 software.

## Results

### Results of the Translation Process

The translation and development of the European Portuguese version of YPRSRS (EP-YPRSRS) involved four procedures based on the guidelines for translation and cultural and linguistic adaptation proposed by Beaton et al. [20]. First, two different translators translated the original YPRSRS version into the European Portuguese version. Second, a meeting was held with two speech therapists and one otorhinolaryngologist to analyze the translations and prepare the preliminary version of the instrument. In the third stage, the initial version of the instrument was back-translated into English by two other translators whose mother language was English. In the fourth stage, professionals of the dysphagia group used the European Portuguese pre-final version of the instrument (Table 1).

**Table 1:** English and Portuguese version of the scale

English version			
Vallecula			
I	None	0%	No residue
II	Trace	1–5%	Trace coating of the mucosa
III	Mild	5–25%	Epiglottic ligament visible
IV	Moderate	25–50%	Epiglottic ligament covered
V	Severe	> 50%	Filled to epiglottic rim
Pyriform Sinus			
I	None	0%	No residue
II	Trace	1–5%	Trace coating of the mucosa
III	Mild	5–25%	Up wall to quarter full
IV	Moderate	25–50%	Up wall to half full
V	Severe	> 50%	Filled to aryepiglottic fold
**European Portuguese version**			
Valéculas			
I	Nenhum	0%	Sem resíduos.
II	Vestigial	1–5%	Vestígios sobre a mucosa.
III	Ligeiro	5–25%	Ligamento glossoepiglótico visível.
IV	Moderado	25–50%	Ligamento glossoepiglótico coberto.
V	Severo	> 50%	Valéculas preenchidas até ao bordo epiglótico.
Seios Piriformes			
I	Nenhum	0%	Sem resíduos.
II	Vestigial	1–5%	Vestígios sobre a mucosa.
III	Ligeiro	5–25%	Até ¼ da parede posterior da faringe e pregas ariepiglóticas preenchidas.
IV	Moderado	25–50%	Até 1/2 da parede posterior da faringe e pregas ariepiglóticas preenchidas.
V	Severo	> 50%	Seios piriformes preenchidos até à prega ariepiglótica.

### Pre-Rating

Four clinicians experienced in FEES and residue classification evaluated the randomized selected images containing PR (n = 31). The scale reliability was strong, with an elevated intra-rater internal consistency for vallecula (Cronbach’s alpha = 0.982) and pyriform sinus (Cronbach’s alpha = 0.922). Inter-rater reliability was equally significant and high (0.973 < 0.996).

### Rater Characteristics

Rater characteristics are summarized in Table 2. A total of 13 raters participated in this study, one of whom was a speech and language therapist (SLT) and 12 of whom were physicians. The median years of experience using FEES (IQR) were 4.6 (4) for the overall group. Each of the 13 raters was assigned to groups by years of experience using FEES (≤ 5, *n* = 8; > 5, *n* = 5) with a median experience of 3 years (2) and 7 (2), respectively. Each of the 13 raters was also assigned to groups who did not use the scale. (*n* = 11) and raters who use the scale (*n* = 2).

**Table 2:** Rater characteristics

Raters	*n* (%)	Years of experience
		Median (IQR)
Total	13 (100%)	5 (4)
Experience with FEES > 5	5 (38.5%)	7 (2)
Experience with FEES ≤ 5	8 (61.5%)	3 (2)
Experience using YPRSRS	2 (15.4%)	-
No experience using YPRSRS	11 (84.6%)	-

### Results of the Validation

At the end of the study, there were 90 ratings for each rater per session, comprising 1170 ratings by 13 raters.

Mean score of YPRSRS and distribution of answers by assessment were described in Table 3. The mean scores for vallecula were 3.17 (sd = 1.93) and 3.20 (sd = 1.41) for initial and second assessment, respectively, and 2.89 (sd = 1.38) and 2.87 (sd = 1.38) for pyriform sinus. In terms of distribution according severity classification, the level II was the most reported for both locations and assessments.

**Table 3:** Mean score and distribution of YPRSRS by assessment

Location/Severity Classification	Initial Assessment, *n*(%)	Second Assessment, *n*(%)
Vallecula, mean (sd)	3.17 (1.93)	3.20 (1.41)
I	79 (13.6)	78 (13.4)
II	146 (25.2)	148 (25.5)
III	88 (15.2)	83 (14.3)
IV	130 (22.4)	123 (21.2)
V	137 (23.6)	148 (25.5)
Pyriform sinus, mean (sd)	2.89 (1.38)	2.87 (1.38)
I	117 (20.4)	113 (19.7)
II	140 (24.4)	158 (27.6)
III	94 (16.4)	81 (14.1)
IV	135 (23.6)	133 (23.2)
V	87 (15.2)	88 (15.4)

For the 13 raters and the images, the average ICC coefficient values for the vallecula (*n* = 585) and pyriform sinuses (*n* = 585) for the initial assessment were 0.974 (95% CI 0.970–0.978) and 0.958 (95% CI 0.950–0.964), respectively. Robust positive correlation results were obtained for both the vallecula and pyriform sinus ICC coefficient values, according to the FEES experience of the study group (> 5 vs. ≤ 5 years; 0.978, 0.9975; 0.972, and 0.947), respectively; Table 4).

**Table 4:** Intraclass correlation

Location	Group/Subgroup	Type of measure	ICC	Kappa^1^ (95% CI)
All	Overall	Average measure	0.967	0.963–0.970
Vallecula	Overall	Average measure	0.974	0.970–0.978
Pyriform sinus	Overall	Average measure	0.958	0.950–0.964
All	experience > 5 years	Average measure	0.977	0.971–0.981
	experience ≤ 5 years	Average measure	0.960	0.954–0.966
Vallecula	experience > 5 years	Average measure	0.978	0.970–0.983
	experience ≤ 5 years	Average measure	0.972	0.965–0.977
Pyriform sinus	experience > 5 years	Average measure	0.975	0.967–0.981
	experience ≤ 5 years	Average measure	0.947	0.934–0.957

^*1*^ *Cohen’s Kappa*

In the initial inter-rater assessment, moderate kappa values were obtained in the overall results, with a value of 0.613 for the vallecula and 0.588 for the pyriform sinus. Kappa values of 0.637 for the vallecula and 0.529 for the pyriform sinus were reported for the group with more experience in FEES (> 5 years). The raters with less experience (≤ 5 years) obtained a value of 0.588 for vallecula and pyriform sinus. According to the experience using YPRSRS, a kappa value of 0.832 for vallecula and 0.856 for pyriform sinus was reported (Table 5).

**Table 5:** Inter-rater agreement analysis (initial assessment)

Location	Group/Subgroup	Number of raters	Kappa(se)^1^	95% CI	*p*
Vallecula	Overall	13	0.613 (0.009)	0.595–0.630	< 0.001
Pyriform sinus	Overall	13	0.558 (0.009)	0.541–0.576	< 0.001
Experience with FEES					
Vallecula	experience > 5 years	5	0.637 (0.024)	0.590–0.683	< 0.001
	experience ≤ 5 years	8	0.588 (0.015)	0.559–0.618	< 0.001
Pyriform sinus	experience > 5 years	5	0.529 (0.025)	0.479–0.578	< 0.001
	experience ≤ 5 years	8	0.588 (0.014)	0.559–0.616	< 0.001
Experience using YPRSRS					
Vallecula		2	0.832 (0.063)	-------	< 0.001
Pyriform sinus		2	0.856 (0.061)	-------	< 0.001

^*1*^ *Fleiss Kappa* *(standard error)*

In the second inter-rater assessment, kappa values were between 0.604 and 0.509 for both locations. Raters with more experience (> 5 years) had a higher kappa value for valleculas and pyriform sinus compared with the group with less experience in FEES (≤ 5 years). According to the experience using YPRSRS, a kappa value of 0.717 for vallecula and 0.714 for pyriform sinus was reported (Table 6).

**Table 6:** Inter-rater agreement analysis (second assessment)

Location	Group/Subgroup	Number of raters	Kappa(se)^1^	95% CI	*p*
Vallecula	Overall	13	0.604 (0.009)	0.587–0.622	< 0.001
Pyriform sinus	Overall	13	0.509 (0.009)	0.491–0.527	< 0.001
Experience with FEES					
Vallecula	experience > 5 years	5	0.704 (0.024)	0.657–0.751	< 0.001
	experience ≤ 5 years	8	0.572 (0.015)	0.542–0.601	< 0.001
Pyriform sinus	experience > 5 years	5	0.605 (0.025)	0.556–0.654	< 0.001
	experience ≤ 5 years	8	0.443 (0.015)	0.413–0.472	< 0.001
Experience using YPRSRS					
Vallecula		2	0.717 (0.078)	-------	< 0.001
Pyriform sinus		2	0.714 (0.078)	-------	< 0.001

^*1*^ *Fleiss Kappa* *(standard error)*

## Discussion

Identifying and quantifying PR severity during FEES is paramount in managing patients with swallowing disorders. Neubauer et al. validate the YPRSRS, an anatomically defined tool for evaluating residue location (vallecula and pyriform sinus) and residue amount (5-point scale from none to severe). The YPRSRS is the most valid and reliable according to a recent systematic review that compares the qualitative and psychometric properties of FEES-based PR scales and a comparison study realized by Messina et al. [15, 21].

This study translated the YPRSRS from English into European Portuguese. The validation demonstrated excellent overall construct validity and intra-rater reliability for both locations (vallecula and pyriform sinus) (Table 4) and a substantial to moderate inter-rater reliability when the raters were grouped with up to 5 years of experience and those with more than five years of experience in FEES. The inter-rater reliability for the two raters using the scale in clinical practice was high for vallecula and substantial for pyriform sinus (Tables 5 and 6).

Compared to the original version, the European Portuguese version achieved similar results. Regarding inter-rater reliability, the German, Turkish, and Italian versions obtained substantially better kappa values than those obtained in the original and Portuguese versions. In Portugal, performing FEES is within the competence of otolaryngologists. The management of dysphagia in the clinical practice of ENT (Ear, Nose, and Throat) is recent and carried out by a few professionals. Consequently, there are few in number or experience; perhaps the values fall short of those found in other versions (Table 7).

The Portuguese version indicates moderate to substantial kappa values for both locations regarding inter-rater agreement. Raters with more experience in FEES had relatively better values. However, the lower inter-rater agreement values could be because the raters did not regularly use the scale. Many of them were residents, and although they collaborated in FEES, they had only recently learned about the scale shortly before the assessment. Only two of the raters used the scale in their clinical practice, and they showed significantly higher kappa values when compared to those who did not use the scale.

**Table 7:** Comparison of intra-rater reliability, inter-rater reliability, and construct validity kappa statistics for vallecula and pyriform sinus across different translations and validations

	Original YPRSRS	Turkish YPRSRS	German YPRSRS	Italian YPRSRS	Messina et al.
Intra-rater reliability					0.91 (± 0.03)
Vallecula	0.957 (± 0.014)	0.9959 (0.0297)	0.963 (± 0.028)	0.95 (± 0.01)	
Pyriform Sinus	0.854 (± 0.021)	0.9959 (0.0297)	0.944 (± 0.051)	0.94 (± 0.01)	
Inter-rater reliability					0.78 (± 0.03)
Vallecula	0.868 (± 0.011)	0.9672	0.928 (± 0.026)	0.81 (± 0.01)	
Pyriform sinus	0.751 (± 0.011)	0.9761	0.938 (± 0.027)	0.78 (± 0.01)	
Construct validity					
Vallecula	0.951 (± 0.014)	-------	0.943 (± 0.033)	0.98 (± 0.02)	
Pyriform sinus	0.908 (± 0.017)	-------	0.947 (± 0.025)	0.98 (± 0.02)	

The authors consider limitations to their study: (1) there are a few dysphagia experts with experience in using residue severity scales; (2) the number of the raters with experience in YPRRS was considerably less than the other; (3) 65,25% of the raters had five or fewer years of experience in FEES; (4) using images selected from videos sometimes reduces their quality, potentially leading to difficulty in classification; (5) the non-use of original images, and (6) used expert ratings as a “construct.”

In the future, the authors intend to promote training on using residue severity scales in FEES and considering these tools as very important for the evaluation and management of dysphagia.

## Conclusion

The validity and reliability of the EP- YPRSRS were confirmed. The authors hope that the European Portuguese version encourages interest in and using severity scales in the endoscopic swallowing assessment and promotes a common language.

## Data Availability

The data underpinning the results presented in this article are available upon request. Interested parties are encouraged to contact the corresponding author for access to the dataset and any supplementary information.
